# Earthworm-Inspired Multimodal Pneumatic Continuous Soft Robot Enhanced by Winding Transmission

**DOI:** 10.34133/cbsystems.0204

**Published:** 2025-03-19

**Authors:** Jianbin Liu, Pengcheng Li, Zhihan Huang, Haitao Liu, Tian Huang

**Affiliations:** Key Laboratory of Mechanism Theory and Equipment Design of Ministry of Education, Tianjin University, Tianjin 300072, China.

## Abstract

This paper presents an earthworm-inspired multimodal pneumatic continuous soft robot enhanced by wire-winding transmission. First, a derived overlapped continuous control law based on multiple peristaltic waves is introduced to effectively improve the motion performance of the robot. Second, by applying the wire-winding transmission method, the extension of one segment is simultaneously transformed into the contraction of other segments, achieving coordinated deformation and making it more similar to real earthworms. In addition, an autonomous obstacle-avoidance control strategy based on contact force sensing is developed to enhance the environmental adaptability of the robot. Based on these methods, an earthworm-inspired soft robot that can perform multimodal movements with autonomous obstacle-avoidance ability and enhanced motion efficiency is developed. A series of experiments including in- and cross-plane crawling, obstacle avoidance steering, and pipeline crawling are conducted to validate the robot’s multimodal motion capabilities. The robot can achieve a speed of 6.65 mm/s (36.0 × 10^−3^ bl/s) during in-plane crawling movement and 1.66 mm/s (8.97 × 10^−3^ bl/s) during pipeline crawling movement. In terms of the in-plane crawling speed, the robot surpasses other robots of the same type. In conclusion, the robot’s multimodal capabilities and enhanced motion efficiency demonstrate superior overall performance, and the robot has good potential for medical and industrial applications.

## Introduction

The applicable scenario range for rigid robots is restricted to unstructured environments [1]. By contrast, soft robots have inherent advantages owing to their superior ability to interact with humans and adapt to complex environments [2,3]. Researchers have turned to nature for inspiration to address practical issues in mechanical technology, develop new machines, and explore new technologies, leading to the emergence of a new discipline—bionics [4]. With the advent of bionics, soft robots based on the principles of animal movements have gained widespread attention. Various categories of bionic robots, such as robotic fish [5], robotic birds [6], and robotic insects [7], have been developed to provide new ideas for research and to solve practical problems [8].

Extensive research has been conducted on crawling animals such as earthworms, caterpillars, and snakes to apply their crawling movements in areas such as medical endoscopy, soil drilling, and surface crawling robots [9–12]. To address specific issues such as pipeline and gastrointestinal (GI) tract inspection, researchers have mimicked earthworms, the most famous annelids capable of continuous multimodal movement on flat surfaces and in underground channels by utilizing the flexibility of their bodies and their ability to generate peristaltic waves along their body length. Earthworm-inspired soft robots have seen widespread development, with key applications involving pipe inspection [13], soil drilling [14], medical endoscopy [15], and surface crawling [16]. Currently, the predominant motion mode of earthworm-inspired soft robots remains relatively singular, which means that they can only crawl on certain surfaces or through pipelines of a certain diameter, reducing the versatility of such robots compared with the multimodal crawling of real earthworms.

**Fig. 1.**
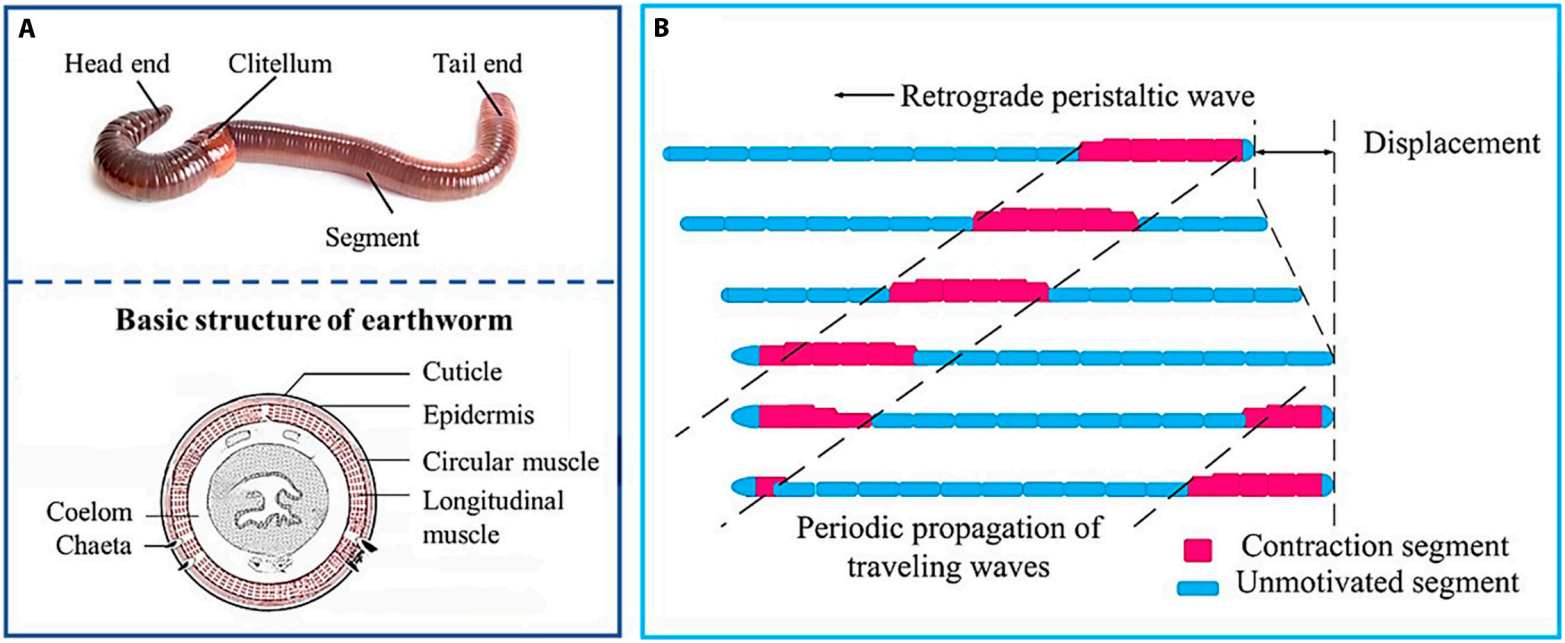
Earthworm structure diagram and movement gait diagram. (A) Structure of an earthworm, (B) peristaltic gait diagram.

Earthworms are terrestrial invertebrates belonging to the phylum Annelida [17]. Earthworms have separate segments with corresponding external and internal layers. This structure makes body segments symmetrical on both sides [4]. As invertebrates, earthworms do not have a skeletal structure but maintain their form through a coelomic cavity filled with tissue fluid [18]. Earthworms achieve locomotion by expanding and contracting individual body segments, and a wave of circular contractions propagates along their bodies from front to back. During peristaltic motion, the direction of wave propagation is opposite to that of self-motion [19]. When burrowing into soil, the internal pressure of earthworm segments increases [19,20]. In addition to the anchoring segments, an earthworm pushes the remaining segments, propelling their front end into the soil. Subsequently, the other segments continue to move forward, becoming new anchored segments [21]. Anatomically, earthworm segments are composed of circular and longitudinal muscles [22] as shown in Fig. 1A. If longitudinal muscles stretch, circular muscles contract correspondingly to ensure constant segment volume, and vice versa. Additionally, friction occurs between the contracting segment and the ground, working as an anchoring segment and allowing the earthworm to extend and move reciprocally. Ultimately, earthworms continue to move forward through this coordinated motion, as illustrated in Fig. 1B.

Most earthworm-inspired soft robots utilize pneumatic- and electric-driven methods, because these 2 driving methods are currently well researched and widely applied. Other driving methods such as tendon-driven [23], magnetic-driven [24–26], and hydraulic-driven [27] methods are also being explored. Among these, pneumatic-driven methods are lightweight, have a fast response, and are environmentally friendly. The pneumatic-driven method, a type of fluid propulsion that is consistent with real earthworms, is currently very mature in the application of earthworm-inspired soft robots.

Currently, only a few earthworm-inspired pneumatic soft robots are capable of performing multimodal motion. A robot developed by Das et al. [28] combines corrugated tubes with airbags to simulate longitudinal and circular muscle movements. It can crawl on flat and rough surfaces and within pipes by changing the inflation pressure and modifying the inflation and deflation modes. Another robot developed by Tang et al. [29] with steering capabilities can move inside pipes and on rough surfaces such as sandpaper. Ozkan-Aydin et al. [30] developed a multimodal robot equipped with a soft, flexible, and extensible body capable of locomotion in most terrestrial environments. Although these multimodal robots may exhibit obvious differences in crawling speed compared with single-modal robots, they still reflect the trend in the research field, mimicking the multimodal motion of real earthworms for better adaptability to multiple scenarios.

Compared to multimodal robots, earthworm-inspired single-modal pneumatic soft robots have been extensively studied owing to their superior motion speed performance, particularly when combined with the wire-winding method. A robot developed by Tirado et al. [31] innovatively employed a dual-tube encapsulation method to achieve motion by inflating and deflating different air pathways. Xavier et al. utilized a wire-reinforcement method and altered the arrangement of fibers outside the telescopic part [32], achieving different actuator deformations, including torsion, elongation, and bending, through variations in fiber winding [33]. In addition, Heung et al. [16] developed a soft robot with wire reinforcement for GI tract inspection. The robot achieved good motion efficiency and crawled through steep slopes without slowing. In conclusion, from previous research, we found that the motion performance of the robot was enhanced through the constraint of the wire-winding transmission method.

This study proposes an earthworm-inspired multimodal pneumatic continuous soft robot to simultaneously achieve multimodal motion and good motion performance. Using the derived overlapped continuous control law (DOCCL) and wire-winding transmission, the robot can achieve a maximum planar crawling speed that surpasses that of other robots of the same type by an order of magnitude. The technical contributions of this study are summarized as follows:

1. A DOCCL based on multiple peristaltic waves is introduced and an earthworm-inspired soft robot is developed, allowing the robot to perform multimodal movements with enhanced efficiency.

2. Through wire-winding transmission, the extension of one segment is simultaneously transformed into the contraction of other segments, achieving coordinated deformation and making it more similar to real earthworms, which further enhances the robot’s motion speed and efficiency.

3. An autonomous obstacle-avoidance control strategy based on contact force sensing is developed, allowing the robot to achieve both in-plane obstacle avoidance and cross-plane motion in the experiment, thereby substantially improving the robot’s adaptability.

## Materials and Methods

### Peristaltic wave model and DOCCL

A peristaltic wave refers to periodic muscular contractions that occur during the movement of crawling robots. This waveform characterizes the movement of the units over time, reflecting the motion processes of such robots. To improve motion efficiency, it is necessary to build a peristaltic wave model and investigate how the control law affects the motion efficiency of earthworm-inspired robots.

The basic assumptions in the modeling process are as follows: All units have the same initial length (*L*_0_) and the same deformation (*∆L*). The starting point for the unit deformation is at the beginning of the cycle *t*_0_. During motion, the sliding of the units can be ignored because the robot moves slowly, and the friction between the body and environment is sufficient to avoid slippage. The deformation (*∆L*) is a value that changes over time, and can be represented by a sine function [19].

To characterize the motion of the robot, the following parameters need to be obtained: the initial length of the segment, *L*_0_; the length of the *i*th segment, *L_i_*; and the number of segments, *SN.* For convenience of description, let the last segment, ${\mathrm{seg}}_{\text{last}}$, serve as the initial origin. Then, the coordinates of the *i*th segment, ${\mathrm{seg}}_{i}$, can be represented as

$${\mathrm{seg}}_{i}={\mathrm{seg}}_{\text{last}}+\sum_{k=i}^{\mathrm{SN}}L_{\mathrm{SN}-k+i}$$ (1)

The motion of each segment of the earthworm-inspired robots can be regarded as the result of the superposition of 2 motions: self-deformation and forward motion. For the *i*th segment, the self-deformation $s_{i}$ can be expressed as:

$$\begin{array}{c} s_{i}\left(t\right)=\left\{\begin{array}{c} \sum_{k=i}^{\mathrm{SN}}L_{i}\left(t\right)+A\;\text{sin}\left(\mathrm{wt}+\varphi_{i}\right),\;t\in \text{extension time}\\ \;\sum_{k=i}^{\mathrm{SN}}L_{i}\left(t\right)+A,\;t\notin \text{extension time} \end{array}\right. \end{array}$$ (2)

where *A* is the maximum value of the segment deformation, w is related to the air pressure frequency, and $\varphi_{i}$ is the peristaltic wave loading phase, which is opposite to the direction of motion and increases backward.

The forward motion can be expressed as ξ⋅A, where ξ is the times of completed segment self-deformation. Therefore, the motion of the *i*th segment of the robot, $f_{i}$, can be expressed as:

$$\begin{array}{c} f_{i}\left(t\right)=\left\{\begin{array}{c} \xi A+A\;\text{sin}\left(\omega t+\varphi_{i}\right)+\sum_{k=i}^{\mathrm{SN}}L_{i}\left(t\right),\;L_{i}\left(t\right)>L_{i}\left(t-dt\right)\\ \;\xi A+\sum_{k=i}^{\mathrm{SN}}L_{i}\left(t\right),\;L_{i}\left(t\right)\leq L_{i}\left(t-dt\right) \end{array}\right. \end{array}$$ (3)

To apply the peristaltic wave model, we first calculate the cycle time *T* of the wave. We assume that *k* complete peristaltic waves pass from the head to the tail of the robot, and each wave has a total of *n* driving segments, and the robot has *r* segments actuating simultaneously, and Δt represents the time it takes for the wave to move from one segment to the next. Finally, the cycle time *T* can be expressed as

$$T=\left\lceil \frac{\mathrm{SN}}{k\left(n-r\right)}\right\rceil \Delta t$$ (4)

where $\left\lceil x\right\rceil =\text{min}\left\{n\in Z\left|x\leq n\right.\right\}$. The total average speed $v_{a}$ under a single peristaltic wave is

$$v_{a}=\frac{\mathrm{SN}\cdot \Delta L}{T}=\frac{\mathrm{SN}}{\left\lceil \frac{\mathrm{SN}}{\left(n-r\right)}\right\rceil }\frac{\Delta L}{\Delta t}$$ (5)

If the robot applies winding transmission, when one segment extends, the other segments contract correspondingly. The velocity $v_{l}$ can be expressed as follows:

$$\begin{array}{c} v_{l}=\frac{\mathrm{SN}\;\cdot \;\Delta L}{T}=\frac{\mathrm{SN}}{\left\lceil \frac{\mathrm{SN}}{n-r}\right\rceil }\frac{\Delta L\;+\;\sum_{\text{spare}}\mathrm{kn}\Delta x_{i}}{\Delta t} \end{array}$$ (6)

where $\Delta x_{i}$ is the contraction of the non-actuation (spare) segments. The relationship between ΔL and $\Delta x_{i}$ is given by the following equation:

$$\sum_{\text{actuation}}\Delta L=\sum_{\text{spare}}\Delta x_{i}$$ (7)

We consider a 4-segment robot and introduce its movements. The segment deformation sequences are listed in Table 1.

**Table 1.** Segment deformation sequence under a single peristaltic wave. Note: 1 represents actuating segment, 0 represents non-actuating segment

Step segment	Step 1	Step 2	Step 3	Step 4
Segment 1	1	0	0	0
Segment 2	0	1	0	0
Segment 3	0	0	1	0
Segment 4	0	0	0	1

**Fig. 2.**
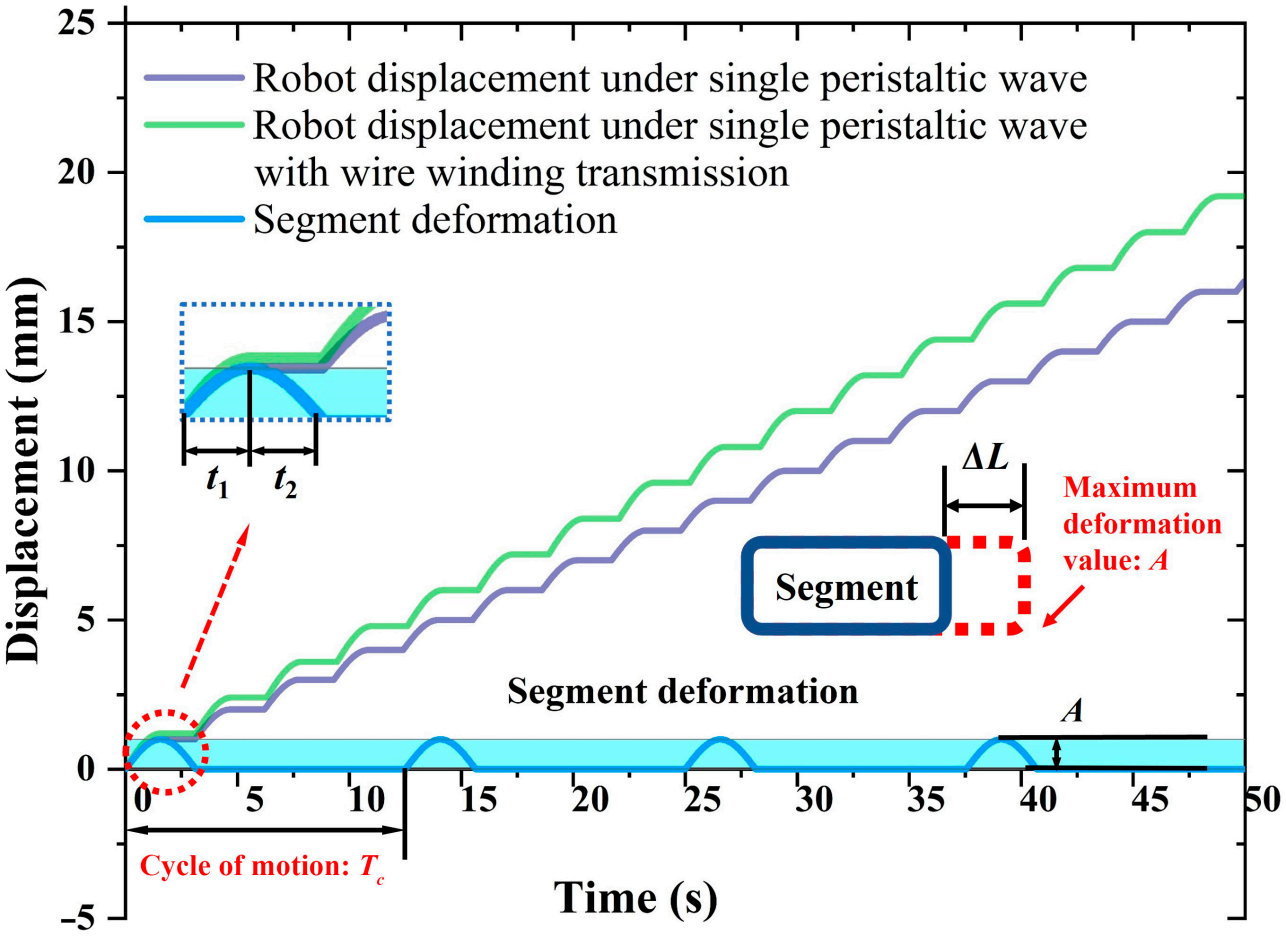
Comparison of displacements under a single peristaltic wave.

As shown in Fig. 2, the segment deformation can be categorized into 2 phases: extension and contraction. In the extension phase (*t*_1_), segment deforms and reaches its maximum length (*A* + *L*_0_). In the contraction phase (*t*_2_), segment recovers its original length *L*_0_. The period of the peristaltic wave is set as *T*_c_. From Fig. 2, it is evident that the wire-winding method has an enhanced effect on the robot motion.

**Fig. 3.**
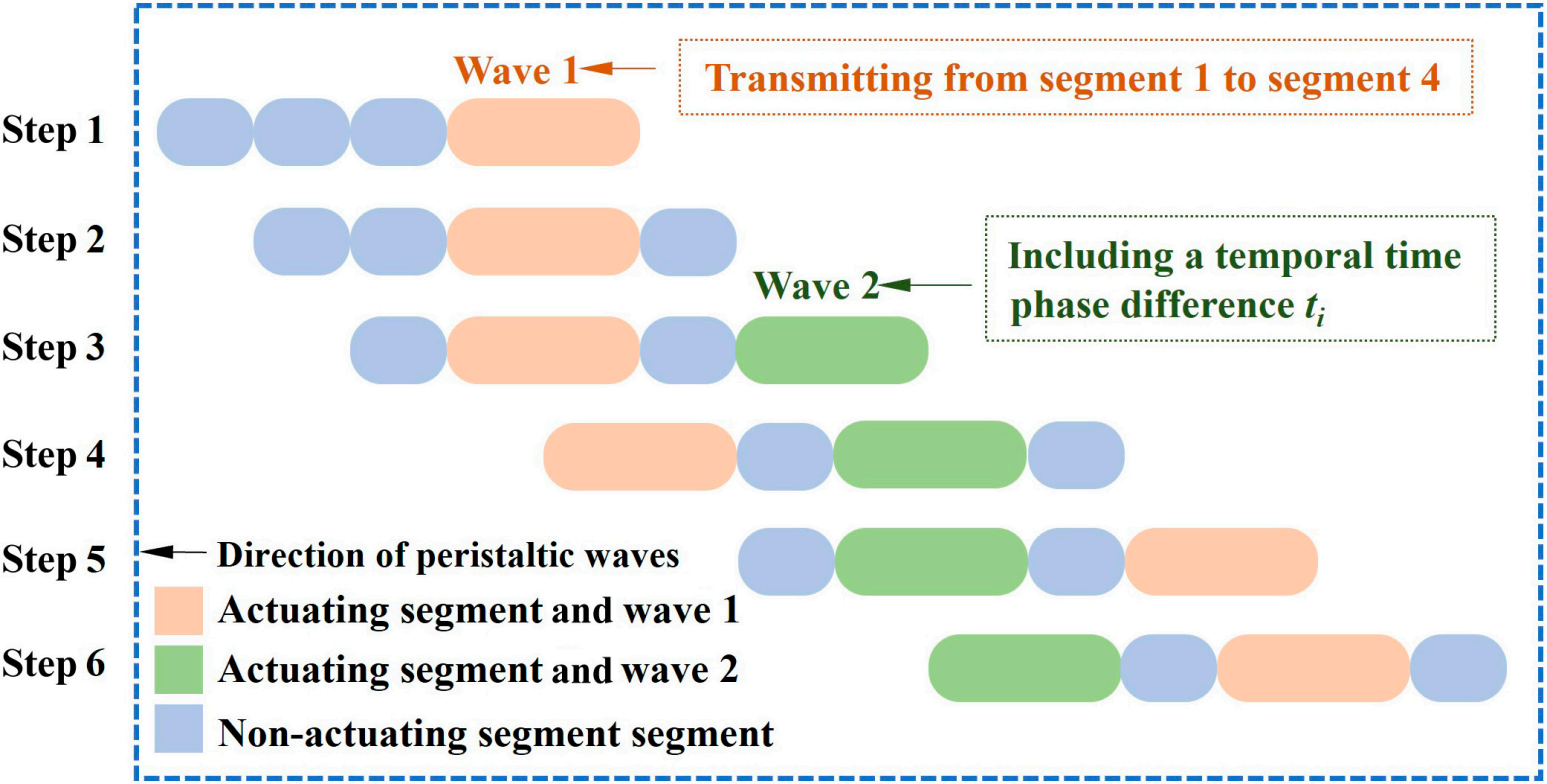
Schematic of double peristaltic waves motion.

In the case of double peristaltic waves, the speed of the robot can be increased to a certain extent. The description is still given using a 4-segment robot. A schematic of double peristaltic waves motion is shown in Fig. 3.

In the case of double peristaltic waves, the period of each wave (cycle of motion) *T*_c_ is the same. Both peristaltic waves are transmitted from robot segments 1 to 4, which are the same as the single peristaltic wave in Table 1. We define *t*_i_ as the interval time between 2 waves, essentially characterizing it as the temporal phase difference (*t*_i_) between the peristaltic waves.

**Fig. 4.**
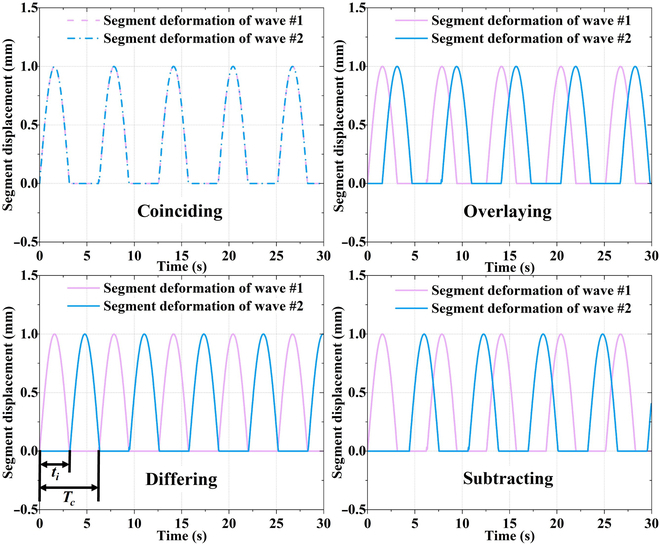
Comparison of 4 conditions under double peristaltic waves.

In general, 4 superposition states are produced under double peristaltic waves, as shown in Fig. 4. In the 4 cases of coinciding, overlaying, differing, and subtracting, the specific values or ranges of the values of their corresponding interval times *t*_i_ are 0, (0, *t*_1_ + *t*_2_), *t*_1_ + *t*_2_, and (*t*_1_ + *t*_2_, *2*(*t*_1_ + *t*_2_)). Owing to the difference in *t*_i_, its velocity can be considered as a superposition of the 2 wave velocities. Assuming that the velocity of the second wave is *v*_sw_, then the total velocity *v_r_* can been written.

When *t*_i_ is between [0, *t*_1_ + *t*_2_], the total velocity *v_r_* of the robot can be written as

$$\begin{array}{c} v_{r}=v_{a}+v_{\mathrm{sw}}=\frac{\mathrm{SN}}{\left\lceil \frac{\mathrm{SN}}{\left(n-r\right)}\right\rceil }\frac{\Delta L}{\Delta t}+\frac{\mathrm{SN}}{\left\lceil \frac{\mathrm{SN}}{\left(n-r\right)}\right\rceil }\frac{\Delta L\;\cdot \;\frac{t_{i}}{t_{1}+t_{2}}}{\Delta t} \end{array}$$ (8)

When *t*_i_ is between (*t*_1_ + *t*_2_, 2(*t*_1_ + *t*_2_)), the total velocity *v_r_* of the robot can be written as

$$\begin{array}{c} v_{r}=v_{a}+v_{\mathrm{sw}}=\frac{\mathrm{SN}}{\left\lceil \frac{\mathrm{SN}}{\left(n-r\right)}\right\rceil }\frac{\Delta L}{\Delta t}+\frac{\mathrm{SN}}{\left\lceil \frac{\mathrm{SN}}{\left(n-r\right)}\right\rceil }\frac{\Delta L\;\cdot \;\left(2-\frac{t_{i}}{t_{1}+t_{2}}\right)}{\Delta t} \end{array}$$ (9)

Clearly, the velocity in the coinciding state ( *t*_i_ = 0) is the same as the velocity of the single peristaltic wave, and the velocity in differing state ( *t*_i_ = *t*_1_ + *t*_2_) is twice the velocity of the single peristaltic wave. In general, this can be verified using the synthesis of the waveforms shown in Fig. 4. If the range of *t*_i_ continues to expand, the latter wave can be superimposed on the former wave, which is similar to the 4 basic states.

**Fig. 5.**
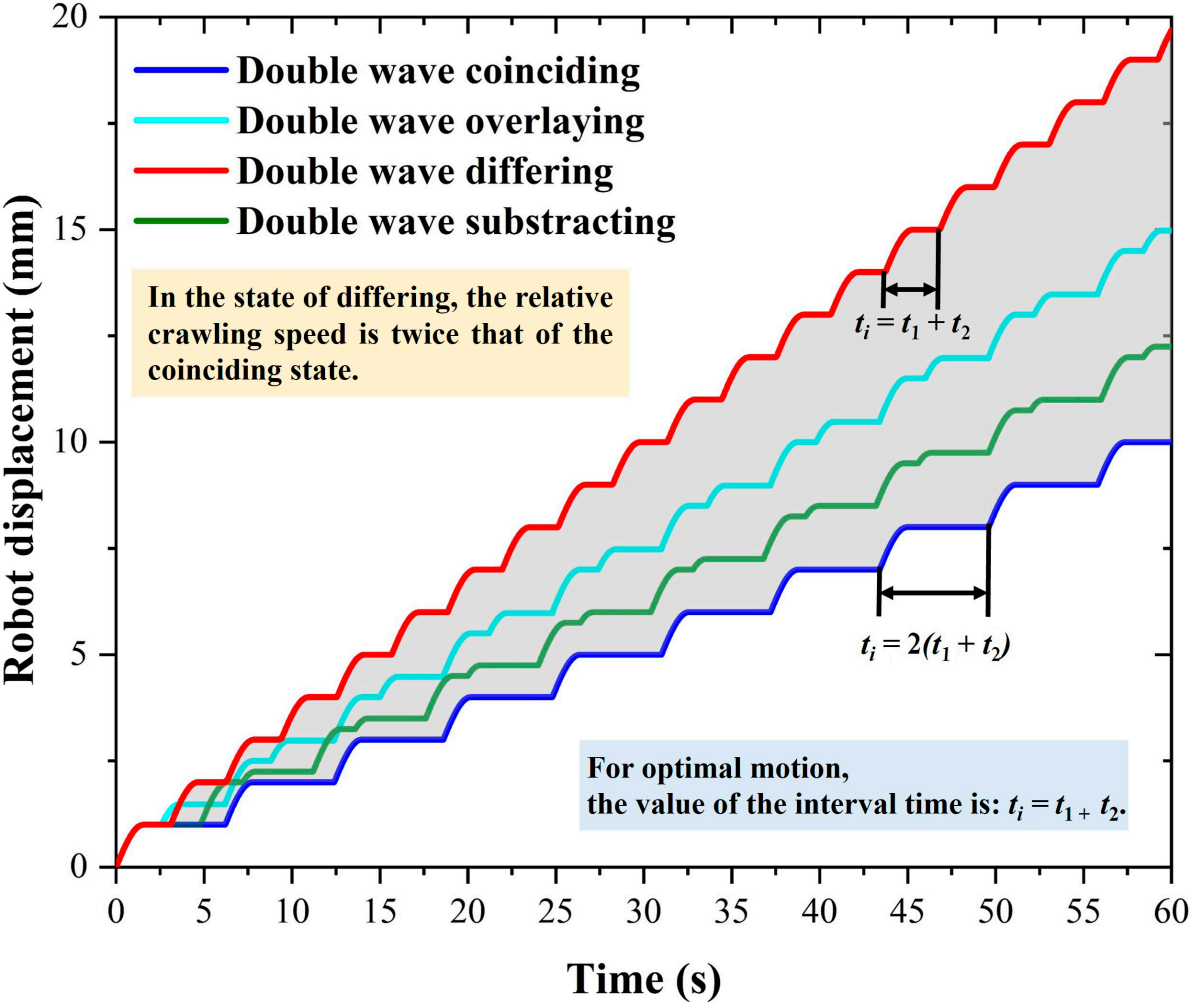
Double-peristaltic-wave 4-state motion waveforms.

As shown in Fig. 5, the motion process of the robot in the 4 basic states is schematically illustrated. During motion, their velocities differ obviously depending on the state of the double wave. In the differing state, the relative crawling speed is twice that of the coinciding state. For optimal motion, the value of the interval time is *t*_i_ = *t*_1_ + *t*_2_. This section demonstrates the necessity of optimizing the interval time *t*_i_ in the subsequent motion control process.

In conclusion, it is evident that the motion efficiency of a robot can be substantially improved by optimizing the interval time. In addition, with wire-winding transmission, the speed of robot movement is greatly increased.

### Design and manufacturing of the robot

The average crawling speed of the robot has been increased using the previously described peristaltic wave model and the DOCCL. Unlike traditional step-by-step motion control, the improved control law achieves continuous earthworm-inspired peristaltic motion. This section discusses the design and manufacturing of the robot segment. The wire-winding method is also discussed.

The segment is made of silicone rubber with a Shore hardness of 50 A, and the material is characterized using the second-order Yeoh constitutive model. It is calculated that *C*_10_ = 0.2043 MPa and *C*_20_ = 0.0014 MPa. In the initial design, we conducted a survey of the commonly used exhaust, ventilation, and gas pipeline diameters. It was found that the main stream diameter of household pipelines is approximately 75 mm. The diameter is fundamental for determining the size of the robot.

**Fig. 6.**
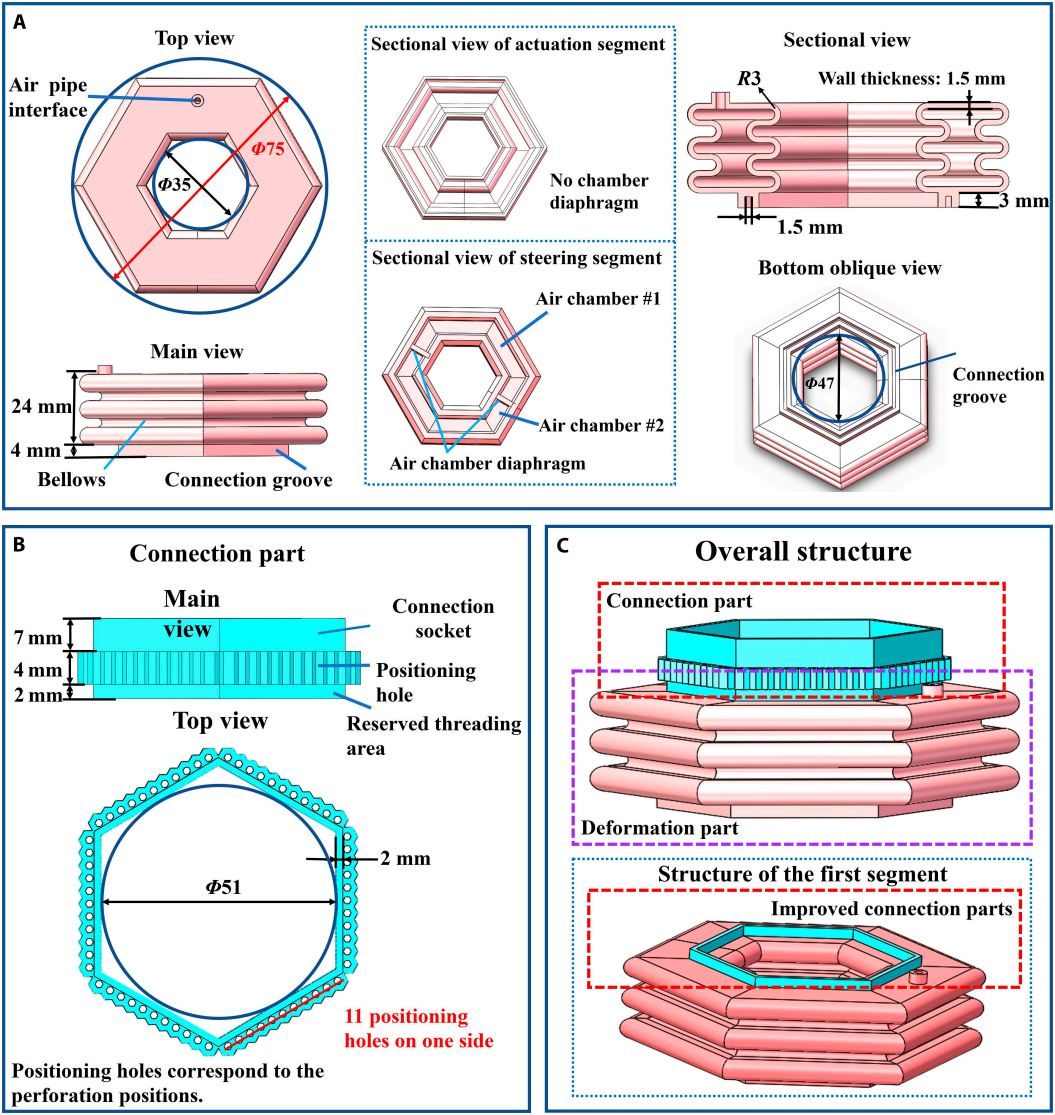
Unit structure of earthworm-inspired soft robot. (A) Deformation part, (B) connection part, (C) overall structure.

The robot segments are identical in appearance. Some segments have an air chamber diaphragm reserved inside that divides the segment into 2 air chambers. Although the segments appear to be the same, the segments with air chamber diaphragms are referred to as steering units; the segments without air chamber diaphragms that drive the robot forward are still called actuation units. The segment structure is shown in Fig. 6. Both units consist of a deformation part and a connection part.

The main structure of the deformation part has a hexagonal cross-section when viewed from the top, with a circumcircle diameter of 75 mm, which is consistent with the diameter of the target pipeline diameter. The side of the deformation part features a continuous corrugated tube with an inner arc diameter of 3 mm and an outer arc radius of 3 mm. The overall wall thickness is 1.5 mm. The total height of the deformation segment in the corrugated tube area is 24 mm (including 3 outer and 2 inner arcs). The bottom of the deformation part has a reserved connecting groove of 4 mm to connect to the subsequent units. The visual differences between the actuation and steering units are the exposed air pipe interfaces, 1 and 2, respectively. The internal difference lies in the presence of the corresponding air chamber diaphragm.

The connection part has a total length of 13 mm and is an overall hexagon with an inner circular diameter of 51 mm. The connection part is divided into 3 sections from top to bottom: a 7-mm connection socket (serving as the threading area), a 4-mm positioning hole, and a 2-mm reserved threading area. Except for the first segment, all subsequent segments need to be connected to the previous segment via a connection part. A connection socket is inserted 3 mm deep into the connection groove of the previous segment and fixed using specialized rubber glue. The wall thickness of the connection part remains at 1.5 mm. During threading, holes are made in the reserved threading area using a perforating needle, and non-elastic threads are passed through, connecting all the units.

In the design process, the unit structure is optimized using Abaqus finite element analysis. We employ an orthogonal experiment to quantitatively analyze the deformation effects and determine the optimal structural size in the design process. It is determined that the deformation part of the segment can achieve approximately 71.5% of the maximum theoretical deformation when inflated under a positive pressure of 25 kPa under the above parameters.

**Fig. 7.**
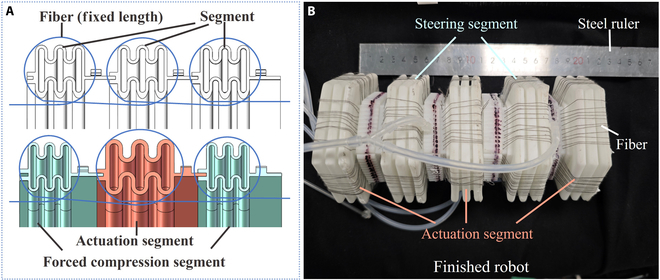
Unit threading and deformation principle and finished earthworm-inspired soft robot. (A) Unit threading and deformation principle, (B) the finished earthworm-inspired soft robot.

In this robot, a non-elastic black cotton fiber wire with a diameter of 0.8 mm is threaded into the robot through the inner winding. The threading and deformation principles are illustrated in Fig. 7A. Eleven threads are arranged along each side, consistent with the positioning hole locations. As the positioning holes of the segments are all in the connection part, they do not affect the performance of the deformation part. Through the wire-winding transmission constraint, the extension of the actuator segment is simultaneously converted into the contraction of other segments, achieving coordinated deformation and making it more similar to real earthworms.

In the process of determining the number of segments, considering that there are no differences in the appearance of the segments and the deformation characteristics of positive pressure driving, the distinction between the actuation and anchoring functions of the segments is no longer made in this robot. Segments that undergo deformation under positive pressure can achieve both axial and radial expansion and complete anchoring. Therefore, to ensure the minimum requirements of the double peristaltic wave model, the number of actuation units is limited to 3, and the number of steering units remained at 2. The total number of units is 5. The finished earthworm-inspired soft robot is illustrated in Fig. 7B.

### Autonomous obstacle-avoidance control strategy of the robot

**Fig. 8.**
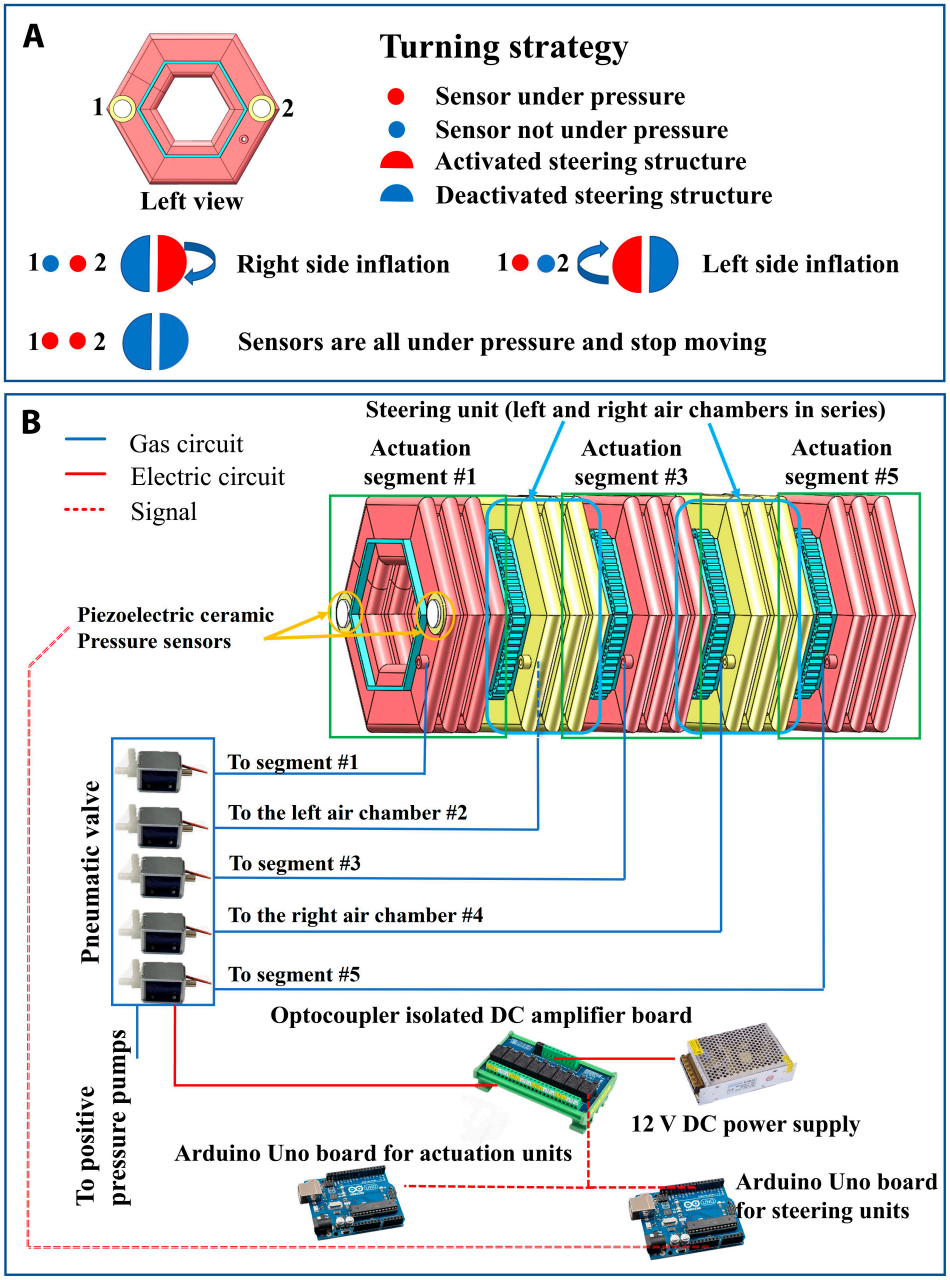
Autonomous obstacle-avoidance strategy of the robot. (A) Obstacle-avoidance strategy, (B) control system.

During the motion process, an autonomous obstacle-avoidance control strategy based on contact force sensing is achieved using piezoelectric ceramic pressure sensors. When the robot encounters an obstacle, the piezoelectric ceramic sensor will detect the pressure signal and convert it into an electrical signal that can be sensed by an Arduino Uno board. The electrical signal is directly proportional to the magnitude of the pressure. The autonomous obstacle-avoidance control strategy for the robot is illustrated in Fig. 8.

Throughout the motion of the robot, the pressure induced by movement vibrations produces unavoidable electrical signals from the pressure sensor. Because it is unnecessary to determine the specific pressure value, and only essential to determine whether the signal is caused by the pressure generated by the robot’s movement vibrations or by the obstacle in front of it, a threshold is set. This threshold is determined through multiple simulations of forward crawling of the robot. The difference in values between obstacles and vibrations is one order of magnitude, and the threshold has not caused judgment errors in the 10 simulations, indicating the feasibility of this method.

In the obstacle-avoidance experiment, when the sensor signal value on one side exceeds the threshold, it indicates that the robot has encountered an obstacle. In response, the controller sends a signal to the steering segments and the robot starts steering to the other side. The controller records the time from the starting point to the finishing point of the steering action as duration time *t*_d_. The robot then performs a reorientation operation to maintain the original direction, and the time required for reorientation is consistent with the duration time*t*_d_, ensuring that the robot advances in the originally intended direction.

Because the steering function is selectively opened and closed during movement, while the straight-line function runs continuously, 2 Arduino Uno boards are chosen and connected to computer. This arrangement allows real-time detection of pressure and multithread control. The other components used in the experiments are listed in Table 2.

**Table 2.** Other components used in the experiment

Experiment components	Specifications
Positive pressure pump	• China Autostyle Trading Co., Ltd
	• 1,100 W 8 l air compressor
	• Nominal flow rate: 8 l/min
	• Rated exhaust pressure range: 0–0.7 MPa
Positive pressure regulating valve	• China Bailing Pneumatic Technology Co., Ltd
	• IR2000-02BG pneumatic pressure regulating valve
	• Adjustment range: 0–0.25 MPa
	• Capable of being used in conditions below 1 MPa
Digital pressure gauge	• China Meko Sensing Technology Co., Ltd.
	• Measure compound pressure: −0.1–1 MPa.
Miniature 3/2-way solenoid valve	• China Zhirong Vacuum Equipment Co., Ltd.
	• Fa2021B miniature 3/2-way solenoid valve
	• Working pressure range: −50 to +100 kPa
	• Flow rate: 1 l/min

As shown in Table 3, valves 1, 3, and 5 are responsible for controlling the first, third, and fifth actuating units of the robot, respectively. The fifth step of the motion state is the same as that of the initial state, and 4 interval times are required. Therefore, the optimal interval time must be obtained experimentally.

**Table 3.** The opening and closing status of each solenoid valve during one movement cycle

Solenoid valve	Initial state	Step 1	Step 2	Step 3	Step 4
Valve 1	Off	Off	On	Off	Off
Valve 3	Off	Off	Off	Off	On
Valve 5	Off	On	On	On	On

## Results

### In-plane crawling performance

**Fig. 9.**
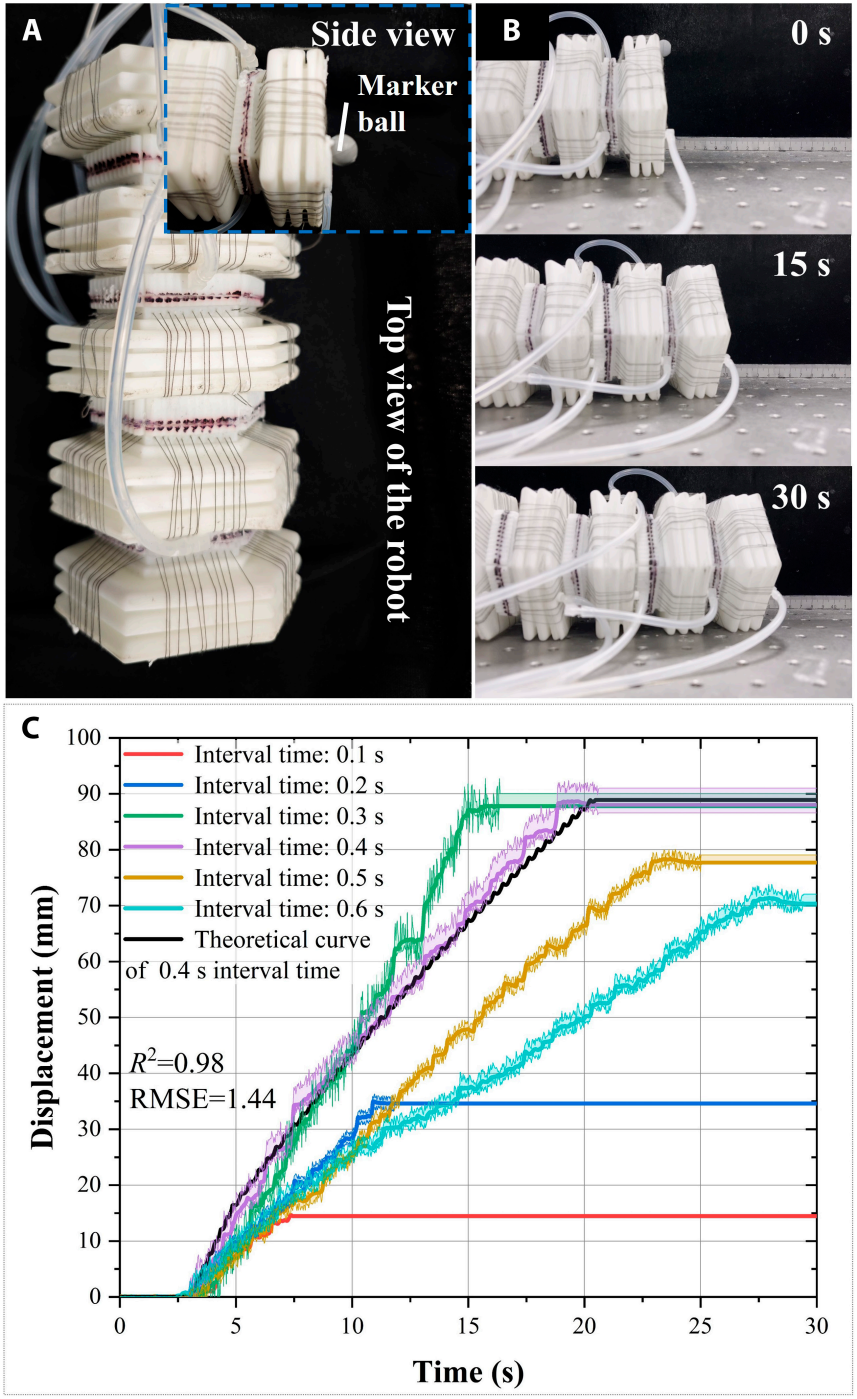
In-plane crawling performance experiment of the robot. (A) Instrument setup, (B) motion process, (C) in-plane displacement–time image.

The robot must undergo an in-plane straight-line crawling performance experiment to obtain the interval time *t*_i_. In this process, a tracking locator is used to track and collect the robot’s real-time position. Marking balls are attached to the front end of the robot. The motion process is illustrated in Fig. 9B. The relationship between the robot motion distance and time is illustrated in Fig. 9C. Each set of variable experiments is repeated 5 times, and the average displacement is calculated. When the interval times are between 0.1 and 0.6 s, the motion cycle is between 0.4 and 2.4 s. After 11 cycles, the corresponding average motion distances are 14.46, 34.62, 87.80, 88.02, 77.70, and 70.36 mm, and the motion average absolute velocities are 3.29, 3.93, 6.65, 5.00, 3.53, and 2.67 mm/s. The experimental results indicate that with a 0.3-s interval time and a motion cycle time of 1.2 s, the optimal robot crawling speed is 6.65 mm/s, resulting in a relative crawling speed of 36.0×10^−3^ bl/s. The theoretical displacement curve is plotted using the theoretical double peristaltic wave model; the correlation coefficient is 0.98 and the root mean square error is 1.44.

### Autonomous obstacle-avoidance steering

In the steering experiments, a 0.3-s interval time is used for planar steering. Because the steering segment is divided into 2 air chambers, only 2 piezoelectric pressure sensors are required. From the perspective of the robot, if the right sensor detects pressure exceeding the threshold, the right chambers of both steering segments are inflated, causing the robot to turn left. Similarly, if the left sensor detects pressure exceeding the threshold, the left chambers of both steering segments are inflated, causing the robot to turn right.

**Fig. 10.**
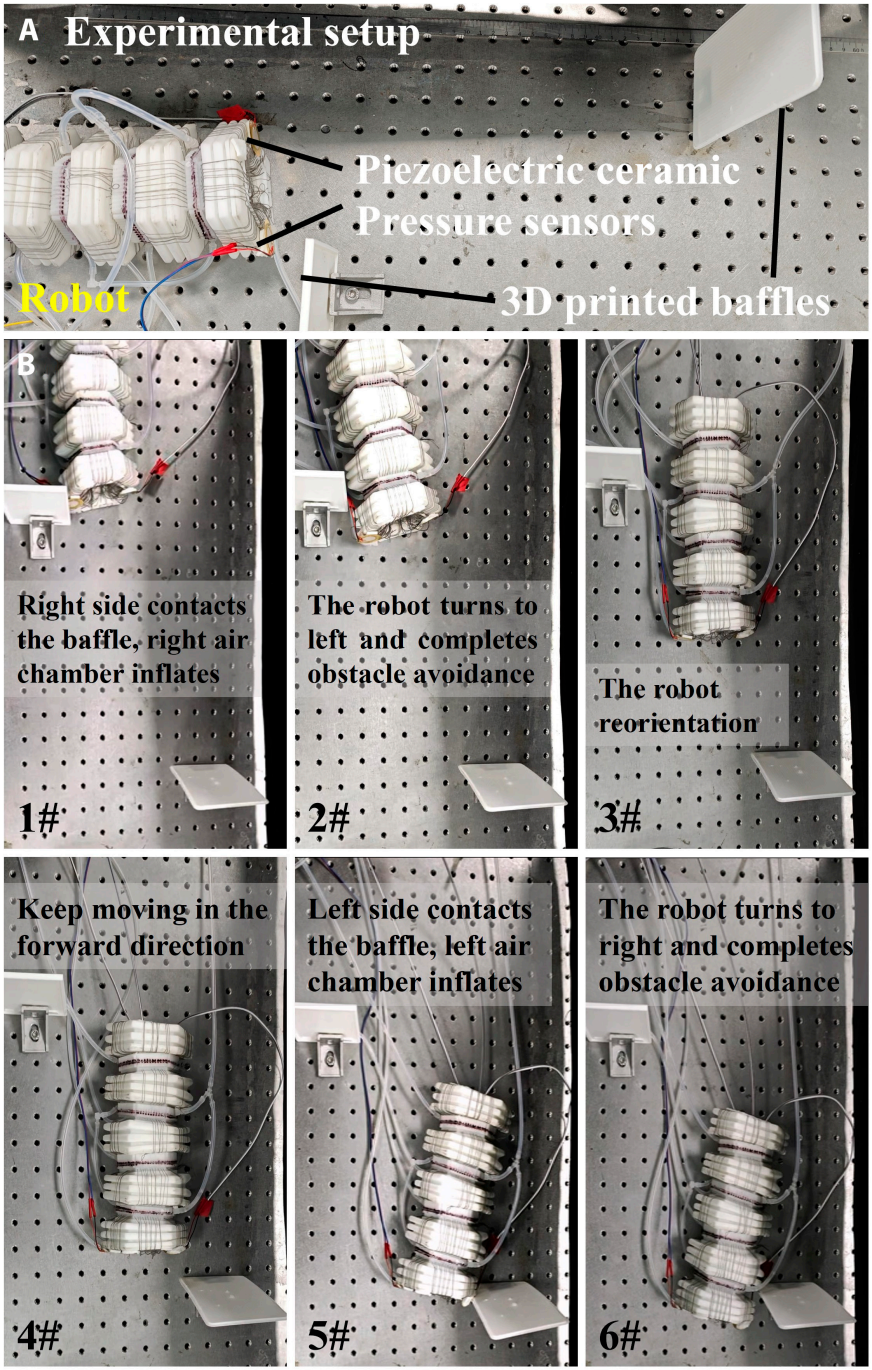
Autonomous obstacle-avoidance steering experiment of the robot. (A) Experimental setup, (B) movement process.

The obstacle-avoidance steering experiment of the robot is shown in Fig. 10B. During the experiment, the robot is placed on a flat platform, and barriers printed via 3-dimensional (3D) printing are placed on both sides of the platform. The right sensor of the robot first contacts the right barrier, sensing pressure exceeding the threshold, and the right chambers of both steering units start to inflate, causing the robot to turn left. After steering and moving forward for 3 s, if neither sensor senses pressure exceeding the threshold, the robot returns to the straight direction and resumes its forward motion. When the left sensor of the robot contacts the left barrier, the sensing pressure exceeds the threshold and the left chambers of both steering structures start to inflate, causing the robot to turn right. After 3 s of motion, if neither sensor senses pressure exceeding the threshold, the robot returns to the straight direction and resumes its forward motion. During the steering experiment, the actuation units are always operational, thereby maintaining the forward motion of the robot.

### Cross-plane crawling performance

**Fig. 11.**
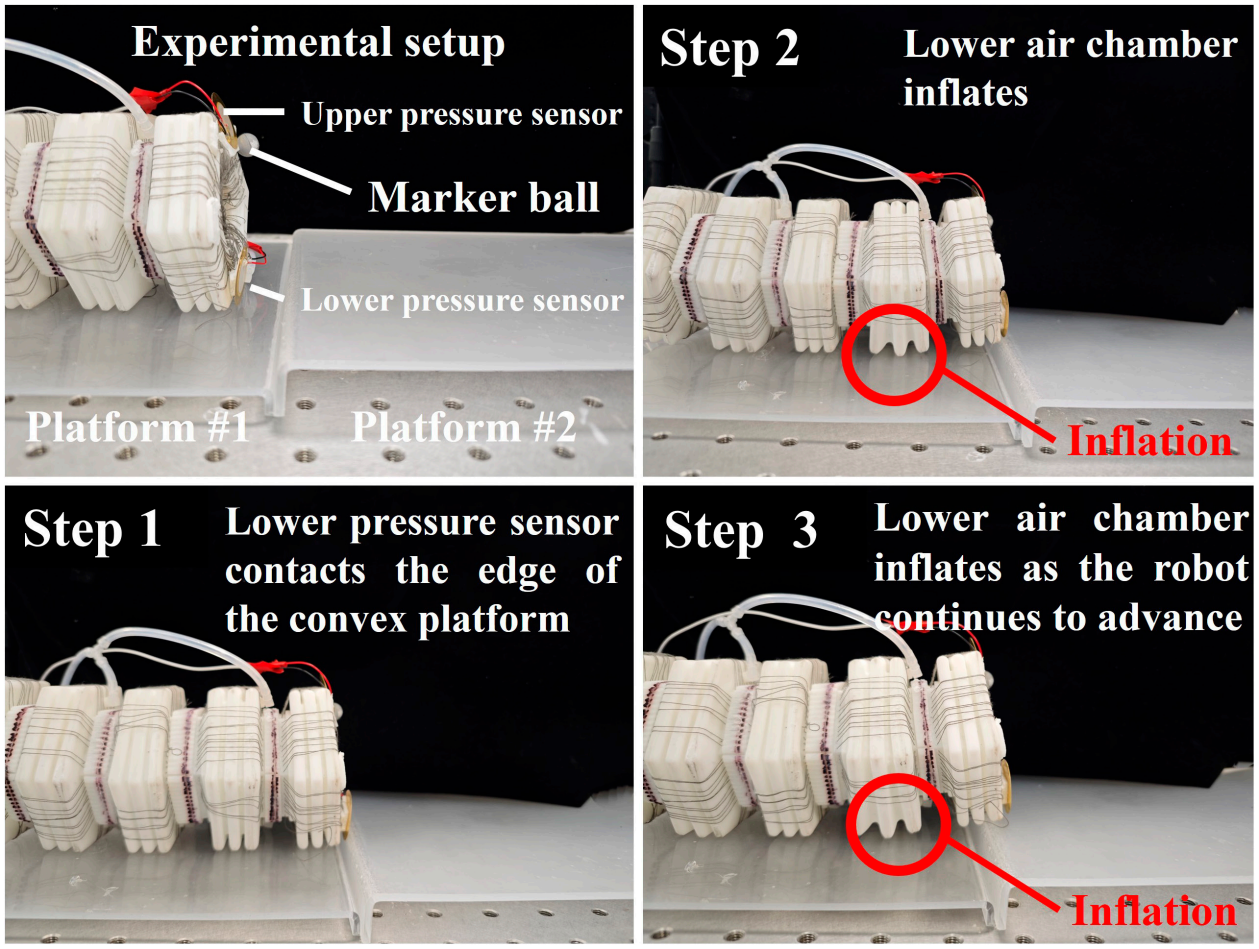
Cross-plane crawling performance experiment of the robot.

By rotating the robot by 90° around its central axis, it can perform cross-plane motion. The motion process is illustrated in Fig. 11. When the front end of the robot makes contact with the edge of the convex platform, the 2 lower chambers are inflated, thereby lifting the robot. It is only necessary to ensure that the last actuation segment is on the previous platform to achieve forward movement. After the first segment of the robot completely crosses the convex platform, the subsequent segments could propel the entire robot forward, because they are still on the surface of the previous platform. As the robot can continue to move forward, it is considered to have completed the cross-plane motion. By conducting tests with different heights of the convex platform, it is found that the maximum height range for the robot to crawl across the plane is 0 to 15 mm.

### Pipeline crawling

**Fig. 12.**
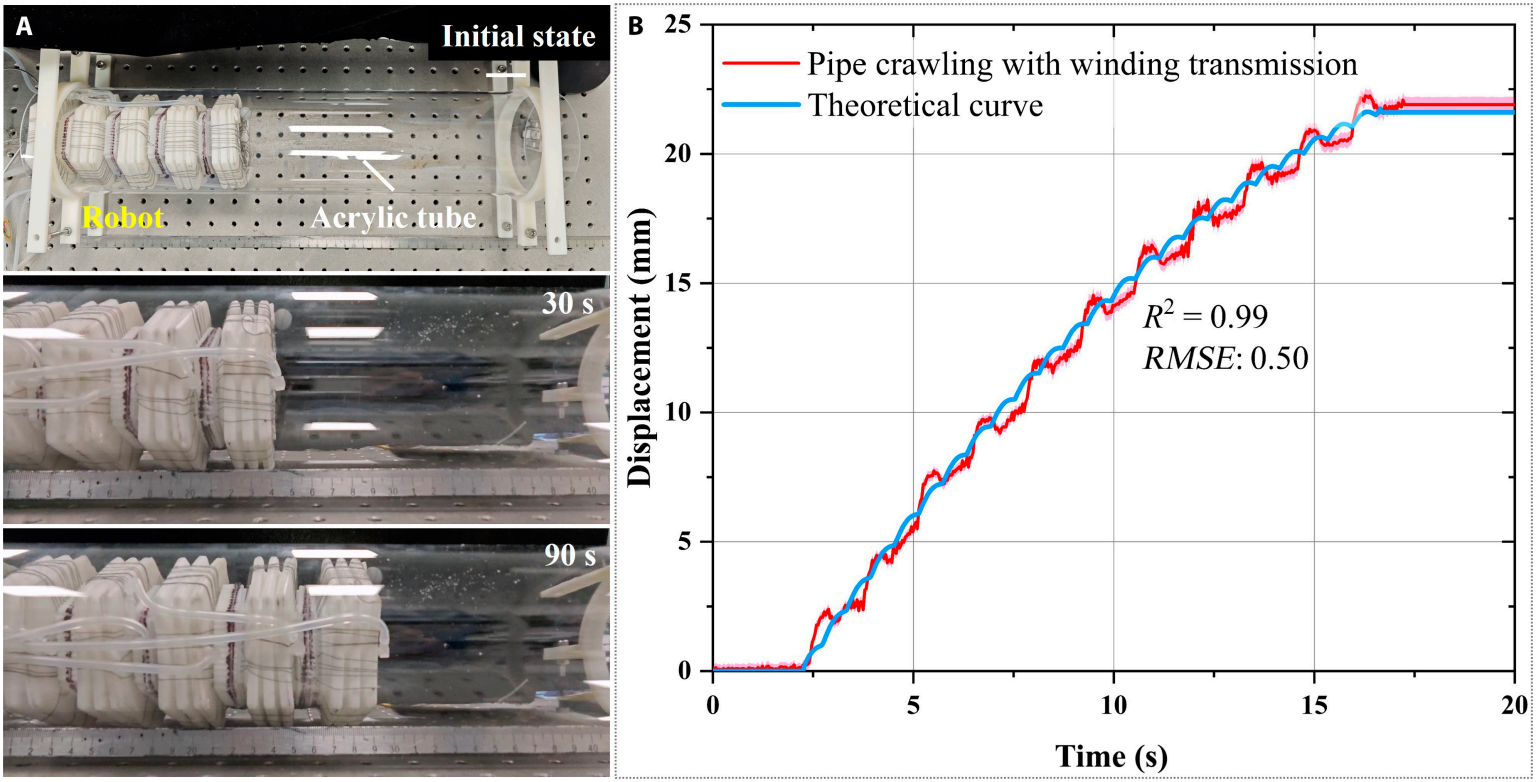
Pipeline crawling experiment of the robot. (A) Pipeline crawling experiment, (B) displacement–time image.

The experiments conducted previously validated the planar straight-line motion, obstacle-avoidance steering, and cross-planar motion capabilities of the robot. In this section, the pipeline crawling capability test of the robot is discussed. A marking ball is installed to monitor the robot’s position in real time. The movement process is illustrated in Fig. 12A. The experiment is repeated 5 times. From the experimental results, it can be concluded that in the case of 11 cycles with an interval time of 0.3 s, and a motion cycle time of 1.2 s, the average displacement is 21.9 mm. Therefore, the average speed is 1.66 mm/s, and its normalized speed is 8.97 × 10^−3^ bl/s.

Comparing the results of pipeline crawling motion with planar motion results, it is necessary to increase the contact area to improve anchoring efficiency. Therefore, in subsequent improvements, optimizing the unit structure by combining rectangles and arcs to form a hybrid cross-section could achieve more efficient pipeline and planar crawling effects. Here, the theoretical displacement curve is plotted using the theoretical double peristaltic wave model with a correlation coefficient of 0.99 and a root mean square error of 0.50.

### Effect of wire-winding transmission

To quantitatively demonstrate the advantages of the robot employing the wire-winding transmission constraint, we design comparative experiments on in-plane crawling and pipeline crawling at optimal interval time. The experimental equipment is consistent with the previously mentioned experiments.

It is important to note that the pressure-bearing capacity of the segment is affected by the absence of wire winding. In the initial inflation tests, it is found that in the case of a deformation part of 24 mm, achieving a change of 12 mm, corresponding to a deformation rate of 50%, requires a pressure decreases from 100 kPa with wire-winding transmission to 20 kPa without wire-winding transmission. To control the variables and achieve identical relative deformations for each segment, the inflation pressure is adjusted to 20 kPa during the comparative experimental process, and the other settings remain consistent. Because there is no wire winding on the segment, the optimal interval time could change. Therefore, displacement–time experiments are still conducted with the interval time of 0.1 to 0.6 s, and the corresponding results are not reiterated here. Ultimately, it is found that the structure still exhibits the optimal cycle at a 0.3-s interval time.

**Fig. 13.**
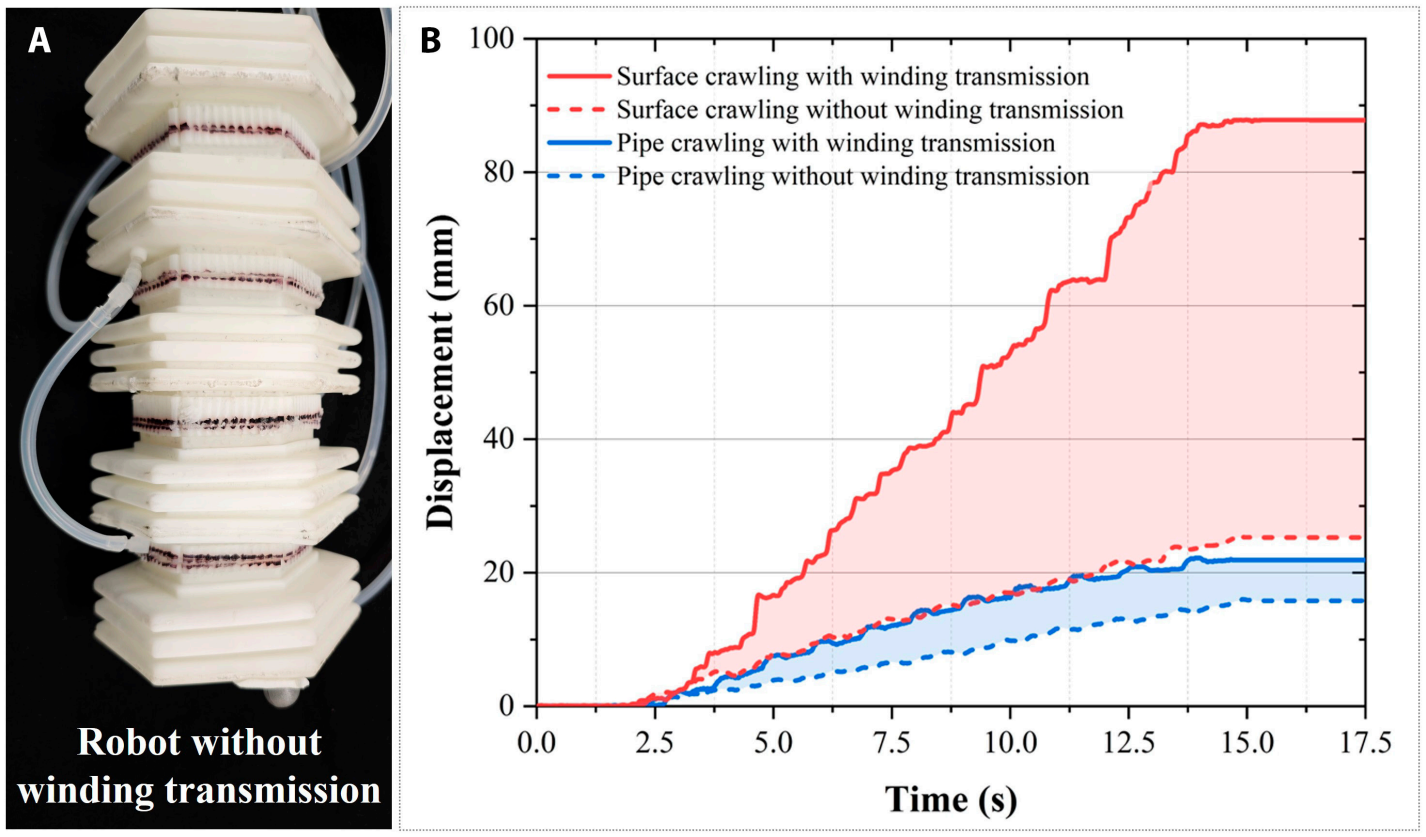
Comparison experiment of wire-winding transmission methods. (A) Overall structure of the robot without winding, (B) performance comparison.

As shown in Fig. 13B, in the state without wire winding, after 11 cycles with a cycle time of 1.2 s, the final planar displacement is 25.27 mm, with an average speed of 1.91 mm/s (10.3 × 10^−3^ bl/s). The in-pipe displacement is 15.77 mm, with an average speed of 1.19 mm/s (6.46 × 10^−3^ bl/s). The shaded blocks in the figure represent displacement reduction in the 2 motion environments.

From this set of experiments, it can be concluded that by relying on the wire-winding transmission constraint, the robot achieves a 249.5% increase in the planar crawling speed and a 38.9% increase in the pipeline crawling speed. This is because when the wire-winding method is adopted, the inflation pressure of the unit at the same deformation is largely increased, resulting in a larger output force of the unit. When moving from rest, the initial acceleration is greater and the slipping phenomenon is less pronounced, producing a larger displacement under equal conditions. However, owing to the nature of the point contact inside the pipeline motion, the increase in the pipeline speed is limited. The wire-winding method not only increases the inflation pressure range and output force of the segment, but also transmits the deformation of a single segment to other segments through winding, further improving the robot’s motion performance and making it more similar to real earthworms.

### Overall performance comparison with other robots by COT

The cost of transport (COT) is a critical indicator when comparing motion efficiency and evaluating the design of earthworm-inspired robots. This represents the power consumed by the robot per unit mass when moving at a unit speed during the motion process. This normalized index characterizes the theoretical deformation capability of the robot and the quality of the related structural design. In this study, the formula used in Kandhari et al. [34] is used to calculate this index for robots of the same type.

$$\begin{array}{c} \begin{array}{c} \mathrm{COT}=\frac{P_{w}}{v_{\text{ideal}}}>\\ \frac{1}{\vartheta }\frac{E_{l}}{E_{\text{c}}}\left[\frac{n_{A}{\left(m+q\right)}^{4}}{2\omega m\left(m+2q\right)}{\left(\frac{R_{s}}{L}\right)}^{-3}+\frac{2\pi n_{A}^{2}}{\omega m\left(m+2q\right)\left(n_{A}-\omega \left(m+q\right)\right)}\frac{R_{s}}{L}{\left(\frac{R_{s}}{h}\right)}^{2}\right] \end{array} \end{array}$$ (10)

where the ratio of power to velocity and weight is given by $P_{w}/v_{\text{ideal}}$, where $P_{w}$ is defined as the ratio of power to weight and $v_{\text{ideal}}$ is the ideal speed. ϑ is Poisson’s ratio, $R_{s}$ is the radius of the unit (body segment), *L* is the length of the unit (body segment), $n_{A}$ is the total number of moving units (body segments) in the robot, $E_{c}$ is the circumferential modulus of elasticity, $E_{l}$ is the Young’s modulus for axial deformation, ω is the number of waves propagating along the length of the robot, *m* is the number of actuation units, q is the number of segments bridging between a pair of moving segments, and *h* is the thickness of the unit.

It is noteworthy that, in Eq. (9), the first term within the square brackets represents the cost coefficient of turning and bending, whereas the second term represents the cost coefficient of unit extension and retraction. Because of the lack of an ideal speed for the relevant robots in this study, *COT* comparisons in this paper are calculated using the inequality terms of this formula. Therefore, all data from the literature are compared by calculating the minimum *COT* value (*COT*_min_). *COT*_min_ is only related to the structural dimensions of the unit and parameters of the control method. The final results are then calculated using the logarithm with a base of 10, as listed in Table 4.

**Table 4.** Comparison of performance of earthworm-inspired continuous soft robot

Reference	Steering ability	Length /mm	Mass /g	Surface crawling speed /(bl∙s^-1^)	Pipeline crawling speed /(bl∙s^-1^)	Minimum cost of transport (lg(*COT*_min_))	Year
Ref. [28]	No	350	N/A	3.80 × 10^−3^	2.06 × 10^−3^	4.47	2023
Ref. [29]	Yes	110	N/A	5.36 × 10^−3^	3.00 × 10^−3^	4.87	2020
Ref. [30]	Yes	450	200	6.35 × 10^−3^	9.51 × 10^−3^	5.34	2022
Ref. [31]	No	40	N/A	N/A	7.75 × 10^−3^	N/A	2023
Our robot (wire winding)	Yes	185	310	36.0 × 10^−3^	8.97 × 10^−3^	4.87	2024
Our robot	Yes	185	307	10.3 × 10^−3^	6.46 × 10^−3^	4.87	2024

Table 4 lists the relevant information and performance parameters of existing earthworm-inspired multimodal pneumatic soft robots. The normalized speed is calculated by dividing the absolute running speed by the initial body length of the robot. In terms of absolute speed, the surface crawling speed of existing multimodal robots is generally below 5 mm/s, whereas the surface crawling speed of our robot can reach 6.65 mm/s, demonstrating a higher movement speed. Compared to other robots, the robot developed in this study exhibits better motion performance in surface crawling. Although this robot has not surpassed the speed indicators of the robot developed in Ozkan-Aydin et al. [30] in terms of pipeline crawling speed, it still shows a speed advantage compared to other robots. Through COT data analysis, the *COT*_min_ data of our robot indicate that there is much progress in optimization of robot segment design, but much less when compared to other robots with similar speed and active steering capabilities. In comparison with current earthworm-inspired pneumatic multimodal soft robots, the robot developed in this study has superior overall performance.

## Discussion

This paper presents an earthworm-inspired multimodal pneumatic continuous soft robot enhanced by wire-winding transmission. First, a DOCCL based on multiple peristaltic waves is proposed, effectively improving the motion performance of the robot. Second, by applying the wire-winding transmission method, the extension of one segment is simultaneously transformed into the contraction of other segments, achieving coordinated deformation and making it more similar to real earthworms. Additionally, an autonomous obstacle-avoidance strategy based on contact force sensing is developed to enhance the environmental adaptability of the robot. After optimization, the robot achieves an average speed of 6.65 mm/s (36.0 × 10^−3^ bl/s) during in-plane crawling movement and 1.66 mm/s (8.97 × 10^−3^ bl/s) during pipeline crawling movement. Comparative experiments reveal that the constraint of the wire winding substantially improves the motion efficiency of the robot on both surfaces and pipelines, with increasing of 249.5% and 38.9%, respectively. These results indicate that the robot in this study has superior overall performance compared to current pneumatic multimodal soft robots. By introducing sensors or specially designed actuators into the robot, there is a lot of potential for use in tasks such as pipeline inspection and soil drilling.

In this work, the deformation and connecting parts are fabricated separately and then glued together by hand, which leads to inconsistencies in the unit, resulting in an initial bending of the robot, making the steering motion into certain direction not sufficiently obvious. Moreover, the use of glue connections poses a risk of air leakage, which affects motion efficiency. By slightly changing the design and adopting non-assembled one-piece 3D printing, the aforementioned problems can be overcome.

In the future, it is conceivable to use a combination of circular and rectangular cross-section segments. During planar crawling, the rectangular parts of the segment provide the contacting surface, whereas in pipeline operation, the circular parts of the segment provide the contacting surface, ensuring that there is always good friction between the robot and the environment. This approach ensures the preservation of the planar crawling speed while effectively enhancing the pipeline crawling velocity of the robot.

## Data Availability

All data needed to evaluate the conclusions in the paper are present in the paper and/or the Supplementary Materials.
